# Decoding depression by exploring the exposome-genome edge amidst COVID-19 lockdown

**DOI:** 10.1038/s41598-024-64200-7

**Published:** 2024-06-12

**Authors:** Xavier Farré, Natalia Blay, Ana Espinosa, Gemma Castaño-Vinyals, Anna Carreras, Judith Garcia-Aymerich, Elisabeth Cardis, Manolis Kogevinas, Ximena Goldberg, Rafael de Cid

**Affiliations:** 1 Genomes for Life-GCAT Lab, Germans Trias i Pujol Research Institute (IGTP), Badalona, Spain; 2 Research Group on the Impact of Chronic Diseases and Their Trajectories (GRIMTra), Germans Trias i Pujol Research Institute (IGTP), Badalona, Spain; 3 ISGlobal, Barcelona, Spain; 4 IMIM (Hospital del Mar Medical Research Institute), Barcelona, Spain; 5 Universitat Pompeu Fabra (UPF), Barcelona, Spain; 6 CIBER Epidemiologia y Salud Pública (CIBERESP), Madrid, Spain; 7 CIBER Salud Mental (CIBERSAM), Madrid, Spain

**Keywords:** Genetics, Psychology, Viral infection

## Abstract

Risk of depression increased in the general population after the COVID-19 pandemic outbreak. By examining the interplay between genetics and individual environmental exposures during the COVID-19 lockdown, we have been able to gain an insight as to why some individuals are more vulnerable to depression, while others are more resilient. This study, conducted on a Spanish cohort of 9218 individuals (COVICAT), includes a comprehensive non-genetic risk analysis, the exposome, complemented by a genomics analysis in a subset of 2442 participants. Depression levels were evaluated using the Hospital Anxiety and Depression Scale. Together with Polygenic Risk Scores (PRS), we introduced a novel score; Poly-Environmental Risk Scores (PERS) for non-genetic risks to estimate the effect of each cumulative score and gene-environment interaction. We found significant positive associations for PERS_Soc_ (Social and Household), PERS_Life_ (Lifestyle and Behaviour), and PERS_Env_ (Wider Environment and Health) scores across all levels of depression severity, and for PRS_B_ (Broad depression) only for moderate depression (OR 1.2, 95% CI 1.03–1.40). On average OR increased 1.2-fold for PERS_Env_ and 1.6-fold for PER_Life_ and PER_Soc_ from mild to severe depression level. The complete adjusted model explained 16.9% of the variance. We further observed an interaction between PERS_Env_ and PRS_B_ showing a potential mitigating effect. In summary, stressors within the social and behavioral domains emerged as the primary drivers of depression risk in this population, unveiling a mitigating interaction effect that should be interpreted with caution.

Depressive disorders are a leading cause of healthcare burden and one of the most common mental health conditions^1^. It has previously been reported that increased risk for depressive disorders, their onset, and maintenance is influenced by lifespan environmental stressors such as physical or emotional abuse during childhood, which are strongly associated with the risk of developing major depressive disorders (MDD)^2^. Furthermore, this relationship is complex and potentially changed by the co-occurrence of other composite risks such as genetic liability and its interaction with some of these environmental stressors, as has been shown for an additive effect of the depression PRS and childhood abuse on depression^3^. Genetic studies show that the underlying genetic architecture of depression is polygenic^4^, influenced by many variants across the genome with individually small effects. Polygenic risk scores are widely used as a metric for additive genetic liability of a given trait or disease, and although polygenic risk scores for diagnosis of depression have shown limited clinical utility, they have proven very useful in etiological research^5,6^. Each of these individual genetic and non-genetic factors have relatively small effects and a combination of them occurring at different points in the lifespan needs to coalesce to ultimately shape the outcome^7^. This information is key to identify the best preventative and treatment strategies towards reducing the burden of depression. Nevertheless, it is unclear how environmental and genetic factors interact and the extent to which they contribute to the phenotype.

The phenotypic expression of depressive disorders in the general population ranges largely from mild, common symptoms to severe manifestations of mental disease. Individuals with subthreshold symptoms of depression are at increased risk of a full diagnosis later in life^8^, and studies report a shared genetic liability between severe forms of major depressive disorder and milder depressive symptoms^9,10^. This perspective aligns with the broader understanding of mental health as existing along a continuum, where individuals may experience different levels of well-being and illness rather than the view of depression as a simple binary (i.e., either present or absent). However, categorical thresholds are used to identify clinically relevant depression and it has been proposed that treatment-resistant forms are particularly associated with genetic susceptibility^11^. It is possible that the contribution of environmental and psychosocial sources of variability is most relevant when mild common forms of depression are considered, but less helpful for treatment strategies aimed at reducing the prevalence of severe cases.

The study of non-genetic effects on depression has proven challenging. Exposure to risk factors such as financial burden or unhealthy lifestyle are more prevalent among vulnerable groups and are highly interrelated^12^, which is a methodological limitation in most studies and can lead to bias in the interpretations due to reverse causality. The first outbreak of the COVID-19 pandemic and subsequent lockdowns exposed the global population to a number of these non-genetic risk factors as the undesirable consequences of mitigation measures, providing a unique opportunity to examine the knowledge about these environmental risks, genetic susceptibility and mental health at population level. Environmental factors during the lockdown were observed to elicit differentiated behavioural patterns, as noted by Delgado-Ortiz et al.^13^. Unexpectedly, these patterns were not necessarily categorized as safe or unhealthy, but rather associated to specific vulnerable groups, thereby challenging the interpretation of individual risks, and perhaps to pointing differences in risk-avoiding attitude. Indeed, health shocks influence individual risk aversion^14^ and in the COVID-19 context they have been shown to affect risk attitudes^15^ known to be genetically determined^16^.

Here, genetic and non-genetic factors were explored simultaneously through the exposome approach. The concept of exposome^17,18^ has been referred to as the sum total of all environmental exposures, to represent the vast environmental dimension in a similar manner to the hereditary dimension. This includes environmental factors such as: local ecosystems, lifestyle choices, social factors, life experiences, and the physical environment^19–21^. This approach has been used by our group and others in numerous works and in several domains, such as working life health^18^, chronic diseases^22^ or early-life risk^23^, however its application remains rare in the field of psychiatric disease^24–26^ as the majority of the research is focused on individual candidate exposures^27,28^.

The objective of this study is to examine the role of the modifiable risk factors of depression using the HADS-D scale as outcome, in a cross-sectional analysis of a single population, using a population-based cohort during the COVID-19 lockdowns. The secondary objective is to study the crosstalk between these factors and genetic factors that account for a part of the heritability^4^ through the use of polygenic predictors with the aim of gaining insights into actionable measures. Here, we generated cumulative poly-environmental risk scores (PERS) from socio-economic, lifestyle and wider environmental and health related risks associated with depression during the lockdown. We also explored individual genetic risk via polygenic risk scores (PRS) using stringent and broad definitions of depression to explore the continuum genetic liability spectrum of depression. Finally, we estimated the predictive capacity of the complete model including both PERS and PRS and disaggregated the analyses by previous diagnosis of depression using retrospective pre-pandemic data.

While the PRS, which is derived from genome-wide data, takes a hypothesis-free approach and captures a broader spectrum of genetic variants across the genome, it is important to recognize the inherent hypothesis-driven nature of the PERS. Despite the comprehensive assessment of environmental stressors in the COVICAT study, those included in the PERS are based on prior evidence. However, our approach may overlook other stressors not considered in the assessment, which leaves room for unaccounted factors influencing the phenotype.

Examining the interplay between genetics and the exposome during the lockdown will help gain a better understanding of why some individuals are more vulnerable to depression, while other are more resilient. This knowledge can inform the development of targeted interventions, support systems and public health strategies to help individuals cope with the challenges posed by lockdowns and reduce the burden of depression.

## Methods and materials

### Subjects

All subjects belong to the COVICAT study (COVID-19 cohort in Catalonia). This is a prospective epidemiological study that aims to describe the health impact of the COVID-19 pandemic on the adult population in Spain^29–31^. The COVICAT study includes participants from six different pre-existing ongoing population-based cohorts in Catalonia established before the outbreak and developed following the COVID-19 pandemic. The largest proportion of the participants (88%) were sourced from the GCAT|Genomes for Life Study; the GCAT cohort study includes middle-aged participants (40–65 years of age) who are resident in Catalonia, whose recruitment started in 2015^32^. All GCAT participants have Electronic Health Records linkage and genotypes are available for a subset of participants. For data completeness and homogeneity for this study we retained only those individuals sourced from the GCAT cohort.

Briefly, COVICAT harmonized data of all cohorts after the first wave of the COVID-19 pandemic in Spain in March 2020 and the majority of participants (99.7%) were contacted between 28 May 2020 and 15 August 2020. Data collection was primarily completed on a study portal website. Some complementary telephone interviews for participants unfamiliar with web-based approaches were conducted (6.1%). All participants provided informed consent and ethical approval for the study was obtained from the Parc de Salut Mar Ethics Committee (CEIm-PS MAR, no. 2020/9307/I) and the Hospital Universitari Germans Trias i Pujol Ethics Committee (CEI no. PI-20-182).

Out of the eligible participants who were contacted 10,862 (61.5%) agreed to participate; of these, 10,087 (92.9%) participants completed the interview satisfactorily. In the present report we excluded 34 participants who were interviewed in the autumn (between October and November 2020), when new restrictions were applied. We also excluded participants with any missing information for pre-pandemic mental health disorder, or environmental/lifestyle data (N = 835). Complete records were available for 9,218 participants. Genetic analysis was performed in a sub-sample of 2,442 cases (genotyped sample) with complete available genetic information.

### Outcome

Depression outcome was assessed by symptoms of depression using the Depression Subscale of the Hospital Anxiety and Depression Scale (HADS-D)^33^. The subscale counts 7 items and ranges between 0 and 21. The survey’s digitized format forced the users to respond to all items of the scale before submitting, which prevented missing items as well as missing scores. To examine the association between exposures (such as genetic factors and environmental factors) with depression, we categorized depression in different levels of severity, based on informed HADS-D thresholds, as reported in validation studies in Spanish populations^33^, defining three outcome categories (mild, moderate, severe) as binary variables named: mild depression (HADS-D < 5 vs. HADS-D ≥ 5), moderate depression (HADS-D < 8 vs. HADS-D ≥ 8) and severe depression (HADS-D < 11 vs. HADS-D ≥ 11).

To account for the effect of previous diagnosis of depression (and anxiety), available pre-pandemic Electronic Health Records (EHR) were analyzed. Lifetime diagnosis and pharmacy dispensation through EHR were combined with self-reported diagnosis. Pre-pandemic cases of anxiety and depression were defined by EHR-ICD-9 codes (Depression: 296, 311; Anxiety: 300), self-reported diagnosis by survey (i.e. “had you ever been diagnosed by a doctor”) and pharmacotherapy by EHR-ATC codes (antidepressants (N06A) and anxiolytics (N05B)) dispensed at least 12 times in the last 10 years (Details in Supplementary Table S1). For this study, a pre-pandemic mental health score was calculated by assigning one point for diagnosis (EHR or Self-reported) and one point for medication. The score ranged from 0 = no evidence of pre-pandemic mental health disorders, to 2 = individuals diagnosed and taking medication for mental health disorders. Only cases with a score of 2 were considered positive for pre-pandemic depression/anxiety.

### Exposome and the poly-environmental risk scores (PERS)

The exposome approach was used to capture the cumulative environmental influences after the first wave of the COVID-9 pandemic in several domains: lifestyle, social, environmental and health. Briefly, a survey was used and data were extracted for risk factors including lifestyle, household, social support and health. Then, environmental variables (i.e. exposure to air pollutants and green spaces (including normalized vegetation index (NDVI))) were estimated from the participants’ residential addresses using models developed by the ELAPSE project and MODIS, respectively^31,34^. Finally, cases of COVID-19 were defined by information about a positive test for SARS-CoV-2 infection and COVID-19 hospitalization as described elsewhere^30^. The exposome selection included 18 variables: loneliness, interpersonal conflicts in the household, caregiving, living alone, being unemployed after the first outbreak, struggling to pay the rent/food, physical activity, alcohol intake, current smoker, sleep hours, media exposure, access to outdoor spaces during the lockdown, natural views from the household, urbanization, greenness (normalized vegetation index), air pollution (nitrogen dioxide levels), any chronic disease, and diagnosis of COVID-19. A full description of the assessment methods and distribution of exposure measures are presented in Supplementary Tables S2 and S3.

A high correlation was observed between individual atmospheric pollutants: nitrogen dioxide (NO_2_); particulate matter with an aerodynamic diameter of less than 2.5 μm (PM_2.5_); tropospheric ozone (O_3_) and black carbon (BC). Similarly, percentage of green spaces within a census tract and 300m, 500m and 1000m buffer were highly correlated with the normalized difference vegetation index (NDVI). The variables that measured a very similar exposure and with r > 0.8 were considered a group and one variable was included in the analysis as representative of the exposure in the domain^35^. A final low correlation (r < 0.3) was observed among retained pollutant variables.

Due to the heterogeneity of the exposure variables, we created binary variables for each of the 18 individual risk exposures. Binary variables were generated for each individual exposure, where 0 = absence of the risk factor and 1 = presence of the risk factor. A cut-off of 1 was used for categorical variables. For numerical variables, a cut-off was settled using the lowest 25% of the total sample. Descriptions of the binary variables and cut-off is presented in Supplementary Table S2. Resulting binary variables were grouped.

We initially used an agnostic approach; thus variables were grouped together without any preconceived notions or biases. Correlation measures were used to determine which variables tended to co-vary with each other. Hierarchical cluster analysis, using Ward’s method, was used first to cluster variables (see Supplementary Fig. S1). Then, to ensure that the resulting categories were consistent with interpretability they were grouped into meaningful categories according to previous observations^13,31^. Briefly, living alone, which impacts social support networks, which are crucial during pandemics when individuals may rely on support from others, was grouped with other socioeconomic factors such as caregiving responsibilities, loneliness, interpersonal conflicts, unemployment, and struggling to meet basic needs like rent or food. In the behaviour domain access to outdoor space was categorized within the behaviour domain along with other factors such as physical activity, alcohol intake, smoking, media exposure, and sleep. These behaviours can significantly affect individual health outcomes and responses to the pandemic. Access to outdoor spaces in particular may be influenced by individuals’ perceptions of COVID-19 risks and their behaviours in response to those perceptions. In the environment domain, chronic disease and COVID-19 were grouped within the category with other factors including air pollution, built environment or natural views. Together with the high correlation between air pollution, natural views and built environment, we have reported an association of air pollution and COVID-19 outcomes, possibly due to respiratory health impact^31^ and chronic diseases, known for potentially exacerbating the severity of COVID-19 symptoms further underscoring the interconnectedness of health and environmental factors.

Finally, to summarize the contribution of selected environmental exposures we generated cumulative scores for each domain; the Poly-Environmental Risk Score (PERS). Firstly, all binary exposures were added up to build a total PERS including all non-genetic exposure variables (PERS_Total_), then we also generated three sub-categories of PERS in three domains to facilitate the interpretability of risks: (1) PERS_Soc_ for all social and household-related factors (sum of loneliness, interpersonal conflicts, caregiving, living alone, being unemployed, struggling to pay rent or food), (2) PERS_Life_, for lifestyle and behavioural factors (low physical activity, high alcohol intake, current smoker, low sleep, high media exposure, low access to outdoor spaces), and (3) PERS_Env_, for wider environment and health-related factors (low natural views, high urbanization, low NDVI, high NO2, chronic disease and COVID-19 case).

To support validity of built PERS, we conducted a sensitivity analysis using a known group validity test examining the association between the three distinct PERS and key variables within our cohort. Employing linear regression models, we investigated the relationship between the PERS_Soc_ score and participants’ education levels, the PERS_Life_ and self-perceived health, and the PERS_Env_ score and the deprivation index. Regressions were adjusted for sex to account for potential confounding effects. By conducting known group validity tests we sought to assess whether our derived scores effectively captured meaningful differences in sociodemographic and health-related characteristics thereby establishing the credibility and applicability of our scoring system within the context of our study population.

### Genome and the polygenic risk scores (PRS)

Genotypic information was accessible for a sub sample of 2442 of the COVICAT individuals sourced from the GCAT cohort^32^. In brief, genome-wide genotypes were generated using the Infinium Expanded Multi-Ethnic Genotyping Array (MEGAEx) (ILLUMINA, San Diego, California, USA), then imputed, accounting for a total of ~ 20 M unique autosomal variants^36^. All GCAT participants that passed strict quality control were included. Variants with minor allele frequency (MAF) > 0.001 and imputation quality score (R^2^) > 0.3 were retained for subsequent analysis. All included subjects were Iberian from White-Western European ancestry based on self-reported data and PCs analysis^36^. Genotypes are available at EGA (European Genome-phenome Archive; https://ega-archive.org/) under accession ID EGAD00010001664.

In order to improve the robustness of the approach we considered different dimensions of depression by drawing on multiple sources of depression-related phenotypic data, including different measurement methods and indicators potentially capturing a more comprehensive understanding of the genetic condition and enhancing the predictive power of the PRS instruments, from symptoms to clinical diagnosis. These include lifetime depression (MHQ), quantitative endorsement (up to five) depression phenotypes (‘help-seeking’, ‘self-reported depression’, ‘antidepressant usage’, ‘depression^37^, or ‘hospital (ICD-10)’^38^; broad depression^39^, and several depression measures ranging from minimal phenotyping (using data from questionnaires) to EHR definition of depression (using ICD-10 codes) or strictly defined depression (using an online mental health follow-up^40^). Polygenic risk scores for all phenotypic measures for depression^38–40^ were derived using either the GWAS summary statistic data, or the weight from the PGS catalogue^41^ (Supplementary Table S4). Posterior SNP effect sizes for those phenotypic measures that were derived using GWAS summary statistic data were computed using PRScs^42^. Weights from the PGS catalogue were processed by removing strand ambiguous SNPs and discordant SNP alleles. Then, the cumulative score for 14 polygenic risk scores for each genotyped individual were computed using PLINK1.9^43^. Raw PRS were converted into standardized z-scores to compare odds ratios across analyses. Because of the overlapping dimensions of depression used, and to help the interpretation of the results, pairwise correlations between the PRS of different depression measures and data sources were assessed by computing a Pearson correlation matrix with hierarchical clustering using Ward’s method between all the phenotypic measures (Supplementary Fig. S2).

### Statistical analysis

We conduct regression analysis to investigate the association between various factors and different thresholds of depression (mild, moderate, and severe). To examine the association between exposures and the outcome, we used binary variables based on the HADS-D threshold; mild depression (HADS-D ≥ 5), moderate depression (HADS-D ≥ 8), and severe depression (HADS-D ≥ 11). Backwards feature selection method with logistic regression, with bootstrapping, was done to identify the best features associated with each depression threshold. The selection process was repeated through bootstrap resampling with 1000 iterations to ensure robustness. Multinomial regression analysis were then conducted for each level of severity with the outcome categorized as follows: HADS-D: < 5 indicating no depression, 5–7 indicating mild depression, 8–10 indicating moderate depression, and ≥ 11 indicating severe depression and using the no depression category as the reference. Prediction accuracy was assessed using Nagelkerke’s pseudo R^2^. Given the well-documented sex differences in depressive symptoms all analyses were adjusted for sex and age^44^. Similarly, education level was used as a proxy for socioeconomic status and included as a covariate, given its association with depressive symptoms^45^, as well the first ten principal components (PC). All models presented here includes all individuals with environmental and genetic data (n = 2442).

In addition to multinomial analysis, we first computed the main effect of genetic factors (genome model) and environmental exposures (exposome model) by analyzing the association between the Polygenic Risk Score (PRS) and the Personal Environment Risk Score (PERS) measures (PERS_Total_, PERS_Soc_, PERS_Life_, and PERS_Env_) and depression outcomes (mild, moderate, severe). These models consider the genetic contribution and environmental factors to depression individually. All models were adjusted for covariates to depression outcomes. Secondly, the multinomial analysis was conducted for the G + E (genome plus exposome) analysis. As briefed before, we employed a stepwise regression approach with backward elimination based on the Akaike information Criterion (AIC). Initially, we fitted a model including all covariates, genetic, and environmental factors and then iteratively removed variables that did not contribute significantly to the model, as determined by the AIC. This process was repeated with 1000 bootstraps with resampling to enhance the robustness of the G + E model, and the final model was constructed with the variables that were retained in 60% of the bootstraps. The retained factors resulting from the bootstrapped models were subsequently analysed to provide insights into the stability and reliability of the G + E associations using a multinomial regression to assess the effects of these variables across all three depression thresholds. Third, with the retained PRS and PERS in the second step, we computed a multiplicative interaction model to explore potential interaction effects between genetic and environmental factors. This model, known as GxE (genome by exposome), evaluates whether the combined effects of genetic and environmental factors on depression outcomes are greater or different from the sum of their individual effects. We applied a False Discovery Rate (FDR) correction to the main effects results to control for the possibility of false positive results due to multiple comparisons in a comprehensive way. Significance was stated at FDR-adjusted *p* value of 0.05. Forest plots were generated using the forestplot Python package (version 0.3.3).

### Ethics approval and consent to participate

All research was carried out in accordance with relevant national and European guidelines and regulations. The GCAT study was carried out using anonymized data provided by the Catalan Agency for Quality and Health Assessment, within the framework of the PADRIS Program. The participants provided informed written consent. The research conformed to the principles of the Helsinki Declaration.

## Results

We present the results of the analyses in the following sections. Firstly, we provide a detailed description of the sociodemographic characteristics, the distribution of HADS-D subscale and the exposome risks in our sample. Secondly, we present the results of the regression models examining the main effect of each exposome score (PERS_Total_, PERS_Soc_, PERS_Life_ and PERS_Env_) on depression outcomes (mild, moderate, severe). We introduce here the novelty of grouping environmental factors on cumulative scores; poly-environmental risk scores. Thirdly, we show results of the regression models exploring the main effect of genetic effects. Here we introduce the exploratory use of PRS issued from complementary definitions of depression. Fourth, we investigate Genome-exposome addition (G + E) and Genome-exposome interaction (G × E) models.

### Sociodemographic characteristics and HADS-D scores

The mean age of the participants in the COVICAT study was 54.6 years (SD = 7.1), 59.4% of them were female. Almost half of the sample (46.9%) had a “higher level of education” and 10.3% had a “pre-pandemic mental health diagnosis”. Two subsamples used in the analysis (i.e. Subsample A, participants with genetic data, and Subsample B_,_ participants with confirmed pre-pandemic mental disorder) showed differences in age-gender distribution as well as in educational level compared to the whole sample (Tables 1 and 2). Subsample A, was older with a lower education level, a higher proportion of males, a lower prevalence of pre-pandemic mental health diagnosis and similar levels of depression. Subsample B, was older with a lower education level and a higher proportion of females. All these differences were accounted for in the following analysis.

**Table 1:** Distribution of sociodemographic characteristics and depression (HADS-D) categories in the whole sample and subsample A (sample with genetic data).

	Total sample	Subsample A
	N = 9218	N = 2442
	N (%)	N (%)
Current age (years); mean (SD)	54.6 (7.1)	55.5 (6.7)
Gender; female	5477 (59.4)	1353 (55.4)
Education level		
Primary or lower	998 (10.8)	348 (14.3)
Secondary	3900 (42.3)	1143 (46.8)
Graduate or above	4320 (46.9)	951 (38.9)
Pre-pandemic mental health score		
0 (no diagnosis)	6729 (73)	1691 (69.2)
1 (possible)	1536 (16.7)	469 (19.2)
2 (confirmed)	953 (10.3)	282 (11.5)
Depression (HADS-D)		
Mild depression (HADS-D ≥ 5)	3224 (34.9)	860 (35.2)
Moderate depression (HADS-D ≥ 8)	1496 (16.2)	397 (16.3)
Severe depression (HADS-D ≥ 11)	550 (5.9)	160 (6.6)

**Table 2:** Distribution of sociodemographic characteristics in the whole sample and subsample B (sample pre-pandemic mental health diagnosis).

	Total sample	Subsample B
	N = 9218	N = 953
	N (%)	N (%)
Current age (years); mean (SD)	54.6 (7.1)	55.9 (6.8)
Gender; female	5477 (59.4)	695 (72.9)
Education level		
Primary or lower	998 (10.8)	145 (15.2)
Secondary	3900 (42.3)	463 (48.6)
Graduate or above	4320 (46.9)	345 (36.2)

**Figure 1:**
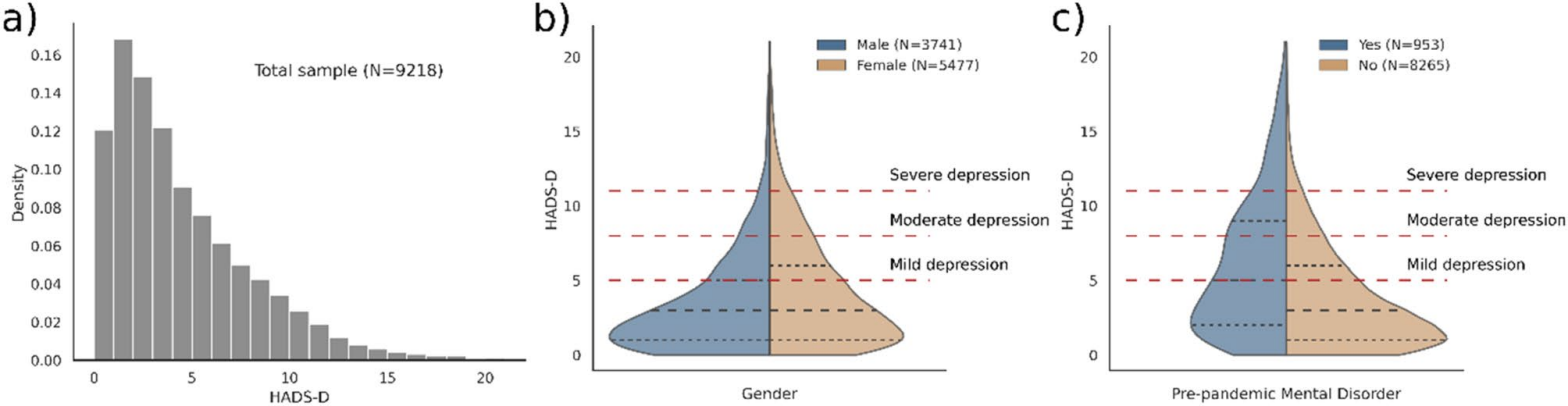
Distribution of total scores of the Depression Subscale HADS-D for (**a**) the total sample, by (**b**) gender, and by (**c**) pre-pandemic mental health diagnosis. HADS-D threshold for levels of depression are depicted with dashed red lines. Similar distribution is observed by gender, with higher HADS-D scores in females than males. Higher HADS-D scores are observed in the participants with previous diagnoses of depression or anxiety.

In the total samples, regarding depression outcomes, there were 3224 (34.98%) participants who scored HADS-D ≥ 5 (mild depression), 1496 (16.23%) participants who scored HADS-D ≥ 8 (moderate depression) and 550 (5.97%) participants who scored HADS-D ≥ 11 (severe depression). Women presented higher depression scores compared with men. Participants with confirmed pre-pandemic mental disorders showed higher depression scores. Distribution of Depression Subscale HADS-D scores are presented in Fig. 1.

### Exposome factors in the full sample

A relevant proportion of the sample reported exposure to known environmental risk factors during the lockdown. For example, loneliness was identified in 75.3% of participants, 9.5% reported interpersonal conflicts in the household, 7.3% lost their jobs after the first outbreak of the pandemic, 8.4% struggled to pay for rent or food, and over 10% had no access to outdoor spaces during the lockdown. A chronic disease was reported by 32.3% of the participants and 4.9% had a positive COVID-19 diagnosis.

Tetrachoric correlation between individual variables revealed imperfect correlations within environmental and socioeconomic variables (Supplementary Fig. S1), with only a few absolute pairwise correlations r > 0.5; *Living alone-caregiving*, *NDVI-urbanization*, *NO2-urbanization*. Ward’s clustering analysis seemed to support the existence of three distinct categories or clusters, particularly for the cumulative scores related to PERS_Soc_ and PERS_Env._ The variable “access to outdoor spaces” was clustered with other environmental risks factors included, possibly due to the high correlation with natural views, but for coherence with other behavioural factors we decided to group this variable with other lifestyle/behavioural factors in the PERS_Life_. Living alone and Caregiving showed a strong negative pairwise correlation and were grouped in a separate cluster, but due to the high correlation they exhibit with other socioeconomic factors, they were considered as such. Finally, variables related to health (i.e. Chronic disease and COVID-19) poorly correlated with most risk factors, were included in PERS_Env_, based on evidence reported by our group correlating COVID-19 and air pollutants^31^. The mean score for estimated PERS were; PERS_Soc_ = 1.01 (0.97 SD), PERS_Life_ = 1.44 (1.04 SD), PERS_Env_ = 1.92 (1.3 SD), and PERS_Total_ = 4.37 (1.92 SD).

We assumed equal weights for the ease calculation, even if it does not fully capture the variability in the impact of individual factors. Reliability of PERS was confirmed by a known group validity test. The known group validity tests yielded statistically significant associations, capturing meaningful variations in education levels, self-perceived health, and deprivation index within the study cohort. Results support the robustness of our composite scores and their further use as instrumental tools. Complete results from the known group validity test are presented in Supplementary Table S5.

### Non-genetic contribution to depression outcomes

**Figure 2:**
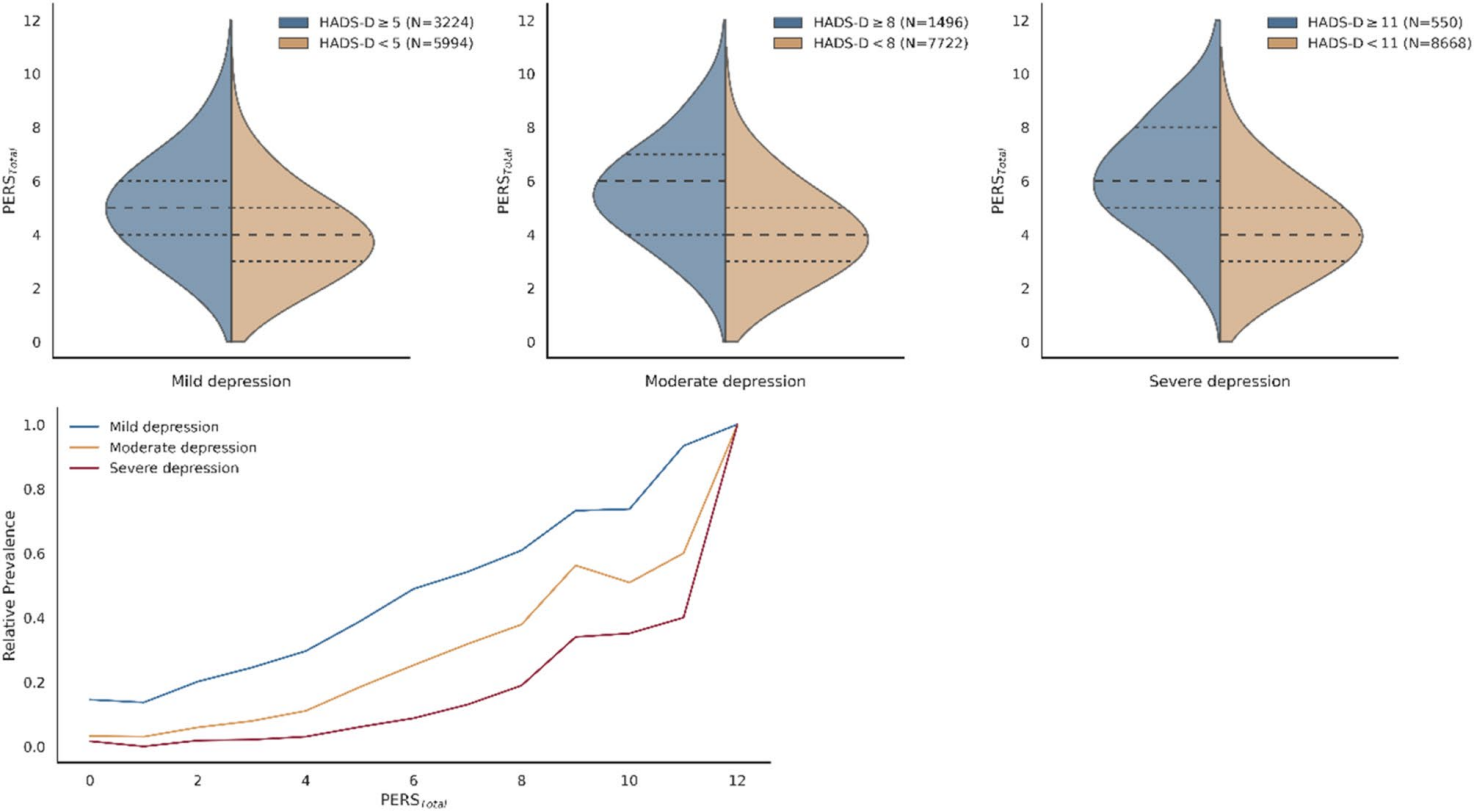
PERS total distribution for each HADS-D threshold. Above, PERS is presented for each of for all three outcomes using HADS-D thresholds named mild depression (HADS-D ≥ 5), moderate depression (HADS-D≥ 8), and severe depression (HADS-D≥ 11), and below, relative prevalence for depression. Relative prevalence was computed as the number of individuals with a HADS-D higher than the threshold for each PERS Total score.

The distribution of depression scores in relation to cumulative PERS at the three considered outcomes (mild, moderate and severe depression) show a consistent increase of depression scores with higher PERS scores and this suggests that higher exposure to environmental stressors may contribute to increased depressive symptoms. Furthermore, there’s a heightened likelihood of individuals experiencing depression across all severity levels as their cumulative exposure to environmental stressors escalates, suggesting that individuals exposed to higher levels of environmental stressors are more likely to experience depressive symptoms, regardless of the level of severity. This trend is consistent for combined score (PERS_Total_) and when we examined each individual domain (PERS_Soc_, PERS_Life_, and PERS_Env_). See Fig. 2 and Supplementary Fig. S3 respectively.

Regression models incorporating exposome measures provided a significantly better fit compared to a null model that only included covariates. The significant associations observed for all PERS with depression for mild, moderate and severe forms (Supplementary Table S6), underscore the potential impact of social, lifestyle, and broader environmental influences on depression health outcomes. Overall, all environmental cumulative scores are associated with a higher depression risk, where the inclusive PERS_Total_ (OR 1.52, 95% CI 1.43–1.63) explained the largest proportion of variance associated with depression regardless of the level of severity (mild: R^2^ = 0.086; moderate: R^2^ = 0.120; severe: R^2^ = 0.140). PERS_Total_ show the highest effect size for severe depression (OR 1.61, 95% CI 1.47–1.77), but the most significant was for moderate depression (OR 1.52, 95% CI 1.43–1.63). Regarding partitioned cumulative scores, PERS_Soc_ showed the largest effect on all assessed levels of severity (mild: OR 1.73, 95% CI 1.57–1.91; moderate: OR 1.87, 95% CI = 1.66–2.10; severe: OR 1.99, 95% CI 1.68–2.36). For moderate depression the social domain is the one that shows the highest effect PERS_Soc_ (OR 1.87, 95% CI 1.66–2.10), as the estimated effect for the lifestyle and behaviour domain (PERS_Life_; OR 1.63, 95% CI 1.46–1.81) and environmental (PERS_Env_; OR 1.36, 95% CI 1.22–1.53) show lower effects. Detailed information for all the models, including the mild and severe depression forms are presented in Supplementary Table S6.

### Genetic contribution to depression outcomes

Polygenic Risk Scores (PRS) associated with moderate depression are defined as HADS-D score of 8 or higher. Two out of all the assessed PRS were found to have a statistically significant increased risk of moderate depression: PRS_B_ (broad depression), (OR 1.18, 95% CI 1.05–1.33, *P* = 2.15 × 10^–2^), a generalized measure of depression and PRS_E1_ (endorsed measures of depression (one)), (OR 1.17, 95% CI 1.04–1.32, *P* = 2.97 × 10^–2^), based on quantitative definitions derived from endorsed measures of depression. Despite the associations observed with moderate depression, none of the assessed PRS were associated with the mild and severe forms of depression. See detailed information for all PRS in Supplementary Table S6. This suggests that the genetic factors captured by these PRS may specifically influence the risk of moderate depression but not the risk of developing milder or more severe forms of the condition.

**Figure 3:**
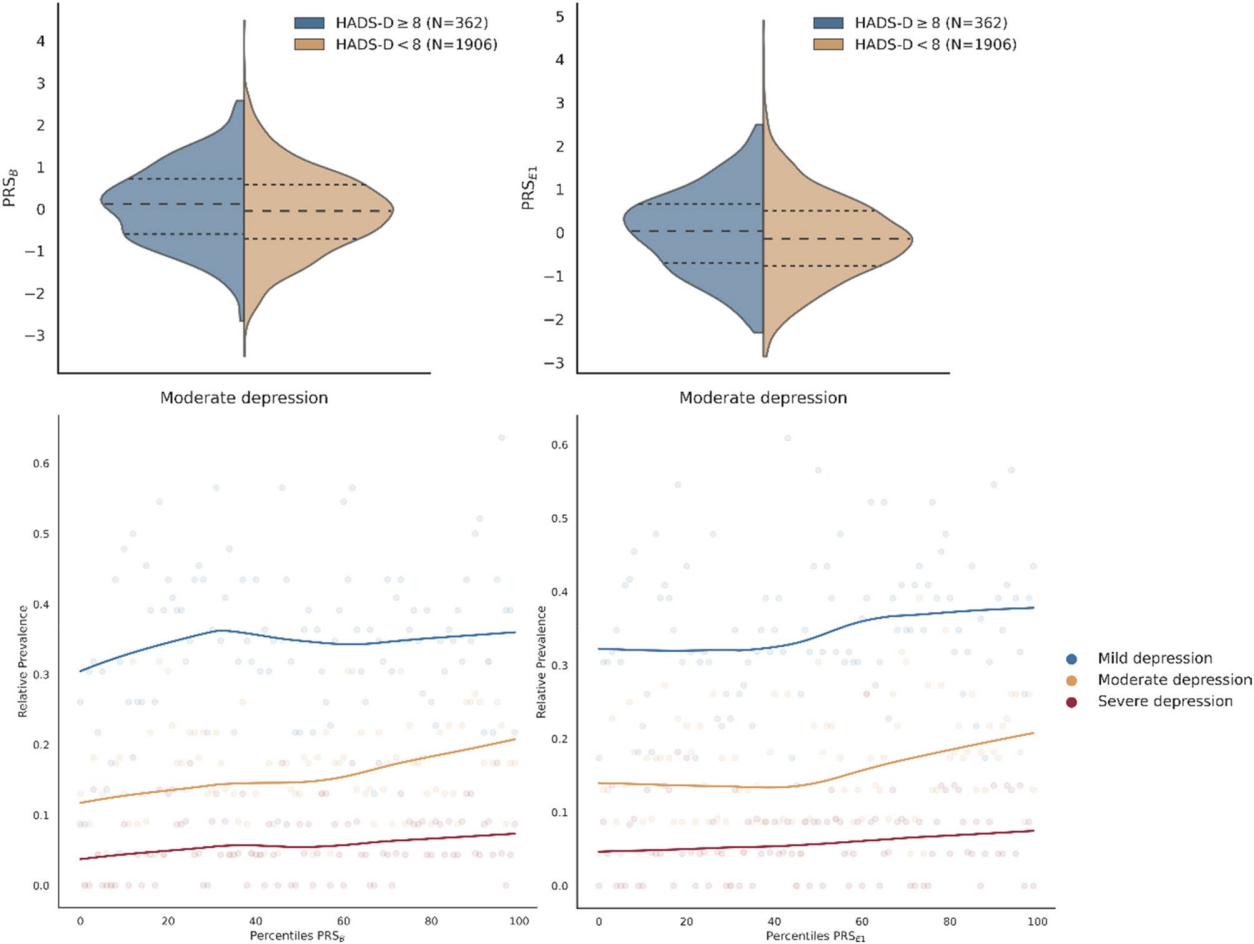
Above, PRS_B_ and PRS_E1_ distributions for the moderate depression outcome. Below, relative prevalence by PRS percentile for all three outcomes (mild, moderate, severe). Individuals were classified according to the PRS percentile and the relative prevalence of individuals with a HADS-D higher than the threshold was computed for each percentile. LOESS curve was fitted to visualize the trend of the relative prevalence by PRS percentile.

HADS-D Scores increase relative to PRS Percentile, suggesting a positive association between genetic predisposition to depression, as captured by PRS, and the severity of depressive symptoms measured by HADS-D scores (Fig. 3). Information for all other assessed PRS are shown in Supplementary Fig. S4.

The effect size of PRSB on HADS-D scores is consistent with reported effects on a broad depression phenotype, as noted in a previous study by Halldorsdottir et al.^3^. This suggests that the genetic factors captured by PRSB are indeed associated with a broad range of depressive symptoms, as previously observed. We also observed a moderate positive correlation (r = 0.55) between PRS_B_ and PRS_E1_, suggesting some degree of shared genetic influence between the two PRS, but it also suggests that there are distinct genetic effects underlying each PRS capturing unique genetic variations associated with different aspects or measures of depression as has been previously suggested by Cai et al.^40^.

### Additive and Interaction risk in severity level of depression

We employed a backwards feature selection method with logistic regression for each different severity level model (mild, moderate, and severe), retaining features that were consistently present in 60% of the iterations for each model (see Supplementary Fig. S5 for detailed description of retained features for all the models). Subsequently, we conducted a multinomial regression incorporating variables retained specifically for each model.

In addition to age, gender and education level, PERS_Soc_, PERS_Life_, PERS_Env,_ PRS_B_, PRS_E2_ PRS_LMDDRec_ and PRS_GP_ were initially retained in one of the models.

**Figure 4:**
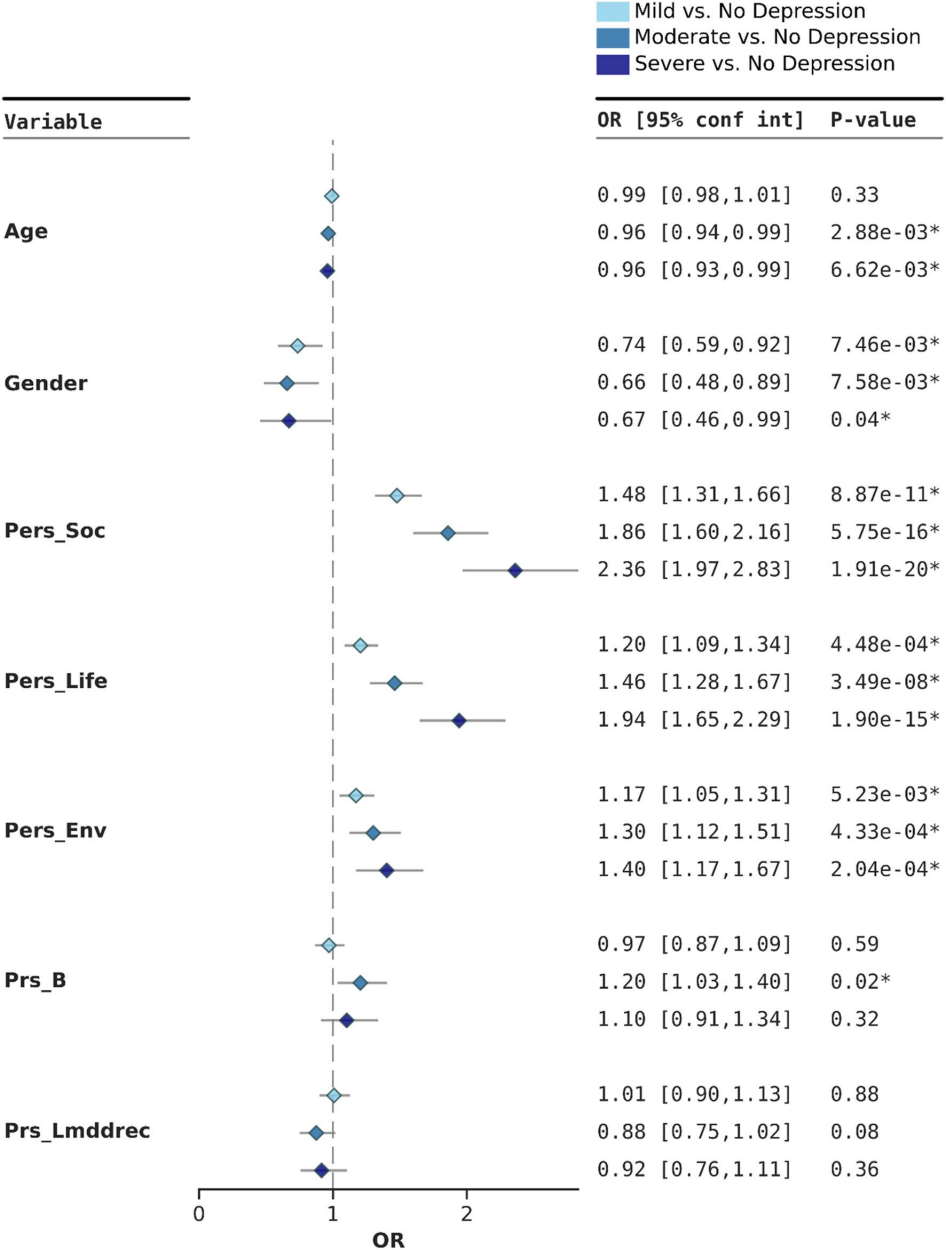
Forest plot depicting the results of the multinomial regression for each depression level including the variables obtained from the backwards selection procedure for the HADS-D threshold for moderate depression.

We further investigated the relationship between environmental factors and genetic risk scores and present results for the moderate depression model (defined as HADS-D score of 8 or higher). This model incorporated age, gender, retained principal components, PERS_Soc_, PERS_Life_, PERS_Env_, and PRS_B_ and PRS_LMDDRec_. The overall variance explained by this multinomial regression model was R^2^ = 0.169. PERS_Soc_, PERS_Life_, and PERS_Env_ scores were significantly associated with depression regardless of the level of severity, suggesting that various aspects of environmental exposures, including social, lifestyle, and broader environmental factors, play a role in influencing the risk of depression. The PERS_Soc_ risk increased significantly as depression severity worsened, specifically we observed a 1.6-fold increase in the PERS_Soc_ OR between mild and severe depression. Specifically, the OR for PERS_Soc_ was 1.48 (95% CI 1.31–1.66) for mild depression and increased to 2.36 (95% CI 1.97–2.83) for severe depression (Fig. 4). Similarly, PERS_Life_, and PERS_Env_ exhibited increasing associations with a 1.6-fold and 1.2-fold increase respectively, between mild and severe depression (PERS_Life_ Mild OR 1.20, 95% CI 1.09–1.34, and Severe OR 1.94, 95% CI 1.65–2.29; PERS_Env_ Mild OR 1.17, 95% CI 1.05–1.31; Severe OR 1.40, 95% CI 1.17–1.67). Regarding genetic scores, we found only a significant association between PRS_B_ and moderate depression (OR 1.2, 95% CI 1.03–1.40) (Fig. 4).

The multinomial regression analysis results incorporating variables retained from the backwards feature selection method for mild and severe level (mild depression, severe depression model) and the analysis with all retained variables combined (any depression model) is presented in Supplementary Figs. S6–S8. Detailed results for each multinomial model assessed is presented in Supplementary Table S7.

Based on these results, we further explored a possible multiplicative interaction effect between the different PERS and PRS_B_. We observed that only the interaction between PERS_Env_ and the PRS attained statistical significance; specifically for mild and moderate depression. Strikingly, the odds ratios for these interactions were consistently smaller than 1, OR 0.87, 95% CI 0.77–0.98, OR 0.84, 95% CI 0.72–0.99, and OR 0.92, 95% CI 0.77–1.12 for mild, moderate, and severe depression, respectively, showing a potential mitigating effect (Supplementary Table S8) and should be interpreted with caution. Sensitivity analysis, in which one variable used to compute PERS_Env_ was omitted at a time, identified NO_2_ as a key factor, the interaction not being significant when NO_2_ was removed from the score. This suggests that NO_2_ may play a driving role in this interaction (Supplementary Table S9).

Finally, to understand the impact of previously diagnosed mental disorders, we distinguish between individuals with and without pre-pandemic mental health conditions, disaggregating by absence or presence of pre-pandemic mental health conditions. The study found significant positive associations for PERS_Soc_, PERS_Life_, and PERS_Env_ scores across all levels of depression severity, regardless of individuals' pre-pandemic mental health status, while polygenic risk scores showed no significant associations, only in the absence of a pre-pandemic mental health diagnosis there was a trend observed for PRS_B_ (Supplementary Table S10). This suggests that environmental factors such as social interactions, lifestyle choices, and broader environmental influences continue to play a significant role in influencing mental health outcomes, irrespective of pre-existing mental health conditions.

## Discussion

Heritability estimates of depression are approximately 30–40%^9^. This leaves ample room for the contribution of non-genetic factors to inter-individual variability of the phenotype. In a single cohort, during the COVID-19 lockdowns, we comprehensively analyzed environmental factors that collectively influence susceptibility to depression, focusing on understanding the impact of the entire exposome^46^. Our approach considered the combined effects of both genetic and environmental factors, and explored the use of cumulative scores of environmental exposures and genetic susceptibility that jointly impact depression.

We identified significant positive associations between non-genetic risk factors and depression levels with a considerable impact of environmental factors on mental health outcomes. After accounting for age, about 16.9% of the differences in depression levels among the individuals in our sample can be explained by PERS_Soc_, PERS_Life_, PERS_Env_, and PRS_B_. In addition, for all three domains, HADS-D Scores increase relative to PERS percentile, suggesting a cumulative effect of multiple environmental stressors on depression risk, and the importance of considering the combined impact.

Specifically, PERS_Soc_ and PERS_Life_ were each associated with a 1.6-fold increase in risk from mild to severe depression, while PERS_Env_ showed a 1.2-fold increase in risk across severity levels. Notably, PERS_Soc_, reflecting social and household-related factors, and PERS_Life_, emerged as pivotal determinants, explaining the major portion of the variance in depression levels. In contrast, wider environmental and health-related factors (PERS_Env_) account for a relatively smaller impact on variance. This underscores the critical role of interpersonal relationships, social networks, and personal well-being in shaping mental health outcomes. Social isolation and social satisfaction play a mediating role in the effect of interpersonal relationships^47^ which increases susceptibility to negative emotions such as depression and anxiety, moreover stress exposure limits the development of healthy interpersonal relationships^48^. The remaining variability in depression levels may be due to other factors not included in our model or random variation. For example, sensitive time windows may model the outcome, as suggested by the strong increase in the incidence of mental health problems in adolescents after the COVID-19 pandemic^49^.

In our study genetics explained a very low proportion of the depression and only PRS_B_ (Broad depression), a generalized measure of depression, was associated with depression, with higher impact on moderate levels of depression compared to milder or more severe forms. These loci may be less specific to the biological underpinnings of clinical depression, while closer to neurobiological mechanisms of symptom onset through susceptibility to exposure to non-genetic factors. The effect size reported here for PRS_B_ (OR 1.2, 95% CI 1.03–1.40) is consistent with reported effects on a broad depression phenotype^3^, suggesting that these genetic factors captured are indeed associated with a broad range of depressive symptoms. This suggests that the depression phenotype captured in self-reported scales is a valid measure of depression and aligns with findings of strong genetic correlation between both measures^39,50^. It’s possible that other genetic factors play a more prominent role in the development, or exacerbation of mild and severe depression, indeed, our analyses indicate that broad definitions of depression showed moderate correlations with more stringent measures of Major Depression Disorder (MDD). However, the results also support the need for a careful consideration of the cut-off scores used to identify clinically relevant individuals in a population-based sample. Substantial discrepancies have been reported between self-assessment using symptoms scales and diagnoses made through strict diagnostic criteria^40^. The cut-off scores of the depression subscale proposed for the HADS-D varied between 5 and 11, where a cut-off score of 11 or higher is considered a strong indicator of clinical depression according to established guidelines^33^. Because stringent definitions of PRS show larger heritability^40^, we expected a stronger association between PRS_LMDDRec_ (Lifetime Major Depressive Disorder (with recurrence)) and higher severity of depression. This was not the case. It is possible that the relatively smaller number of individuals with higher severity limited statistical power.

In our model, we observed broad depression (PRS_B_) and environmental and health dimension stressors (PERS_Env_), shown an interaction with a mitigating effects for mild and moderate depression. From PERS_Env_, air pollution (NO_2_) was identified as the key factor, playing a driving role in this interaction. However, these results are counterintuitive considering previous observations. There is emerging evidence of associations between poor air quality and poor mental health, as well as specific mental disorders^15^. Furthermore, pre-existing long-term conditions appear to deteriorate, requiring more healthcare^51^. We also observed that more severe COVID-19 disease was associated with mental health disorders^29^ and exposure to air pollutants^31^. Overall, this result suggest that complex non-linear relationships may exist, as well unmeasured variables or uncontrolled confounders resulting in misleading conclusions.

Globally, the prevalence of depression has experienced a significant increase after the COVID-19 pandemic^52^. We previously identified that 5% of the participants in the COVICAT cohort with no pre-pandemic history of mental health disorder presented clinically relevant depression two months after the first lockdown^29^. Here we observed that the strong association of poly-environmental scores was independent of pre-existing mental health disorders. Notably, while the statement holds true for cumulative PERS, certain factors seem to individually exacerbate when there are existing mental health conditions, as reported in Goldberg et al.^29^ where Living alone was identified as risk factor for severe depression only for those with pre-pandemic mental health diagnoses, however Household interpersonal conflicts and Financial instability are predictors of severity in people without pre-pandemic mental health diagnosis.

Together, all this evidence challenge a widespread perception that more severe symptoms and/or more strictly defined symptoms of depression are closely related to genetic susceptibility, while social, lifestyle or environmental factors mainly model moderate or non-specific symptomatology^40,53^. Our results show that non-genetic exposures are strong and significant predictors of severe depression and their capacity to shape the mental health of populations should not be underestimated.

Our study has several strengths that contribute to the robustness of the findings, the most relevant being high-quality individual-level data and the low proportion of missing data, the large range of depression phenotypes assessed across phenotypic presentations and the thorough examination of environmental stressors. Furthermore, several important limitations should be considered when interpreting the findings of the study. While the assumption of potential causality is reasonable since exposures precede the outcome, caution is warranted due to the possibility of bidirectional relationships between variables. The presence of healthy bias^54^ and limited representation of ancestry^55^ in the study sample may affect the generalizability of the findings to broader populations, however, this does not invalidate the relationships found between exposures and depression outcomes. Finally, the limited sample size may affect the statistical power, particularly in interaction analyses. Replication studies with larger sample sizes are needed to validate the observed interactions and enhance confidence in the findings.

The findings of the study indeed provide valuable insights into addressing multiple stressors simultaneously to mitigate depression risk and promote resilience^56^. However, it is crucial to interpret cumulative scores and interaction effects cautiously, considering unaccounted factors and potential confounding variables that may influence the observed associations.

### Supplementary Information

Supplementary Figures.

Supplementary Tables.

## Data Availability

The datasets generated during and/or analysed during the current study are available from the corresponding author on reasonable request. Genotypes are available at EGA (European Genome-phenome Archive; https://ega-archive.org/) under accession ID EGAD00010001664.
