# Supplementary material for: Decoding depression by exploring the exposome-genome edge amidst COVID-19 lockdown
Source: Sci Rep. 2024 Jun 12;14:13562. doi: 10.1038/s41598-024-64200-7 (PMC11169603; doi:10.1038/s41598-024-64200-7)
Supplement: Supplementary file 1 — Supplementary Figures. [file 41598_2024_64200_MOESM1_ESM.docx]

**Decoding depression by exploring the exposome-genome edge amidst COVID-19 lockdown**

X. Farre^1,2^, N. Blay^1,2^, A. Espinosa^3,4,5,6^, G. Castano‑Vinyals^3,4,5,6^, A. Carreras^1^, J. Garcia‑Aymerich^3,5,6^, E. Cardis^3,4,5,6^, M. Kogevinas^3,4,5,6^, and X. Goldberg^3,4,7^(*), R. de Cid^1,2^ (*)

**Supplementary materials.**

**
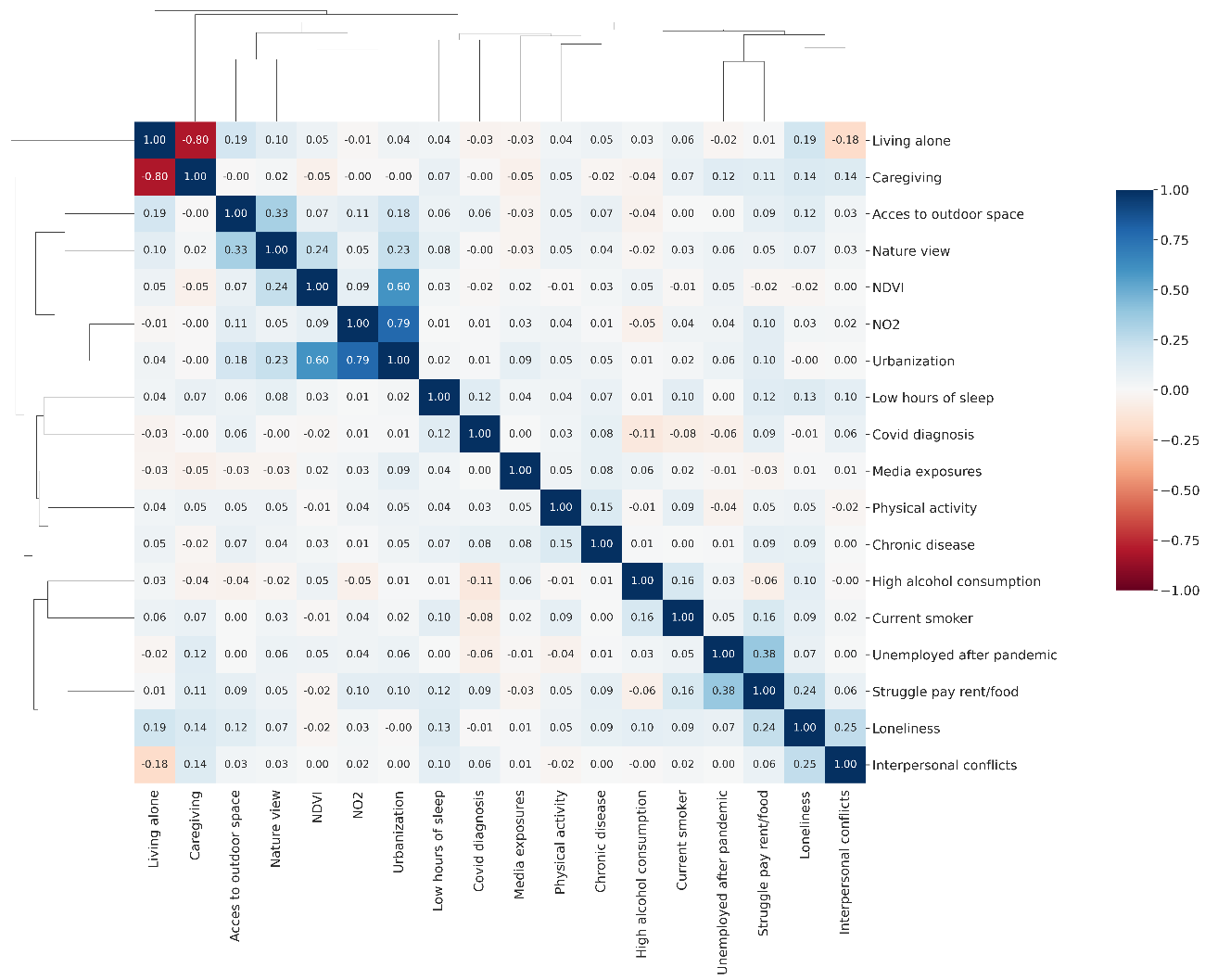
**

Supplementary Figure S1. Tetrachoric correlation between individual non-genetic exposures, including Ward’s clustering. Clustering highlighted the correlation between the factors within the environmental and socioeconomic categories. Although Living alone and Caregiving are classified in an independent cluster due to their strong negative mutual correlation, they have a moderate correlation with other socioeconomic factors. Description of considered variables is shown in Supplementary table 1.


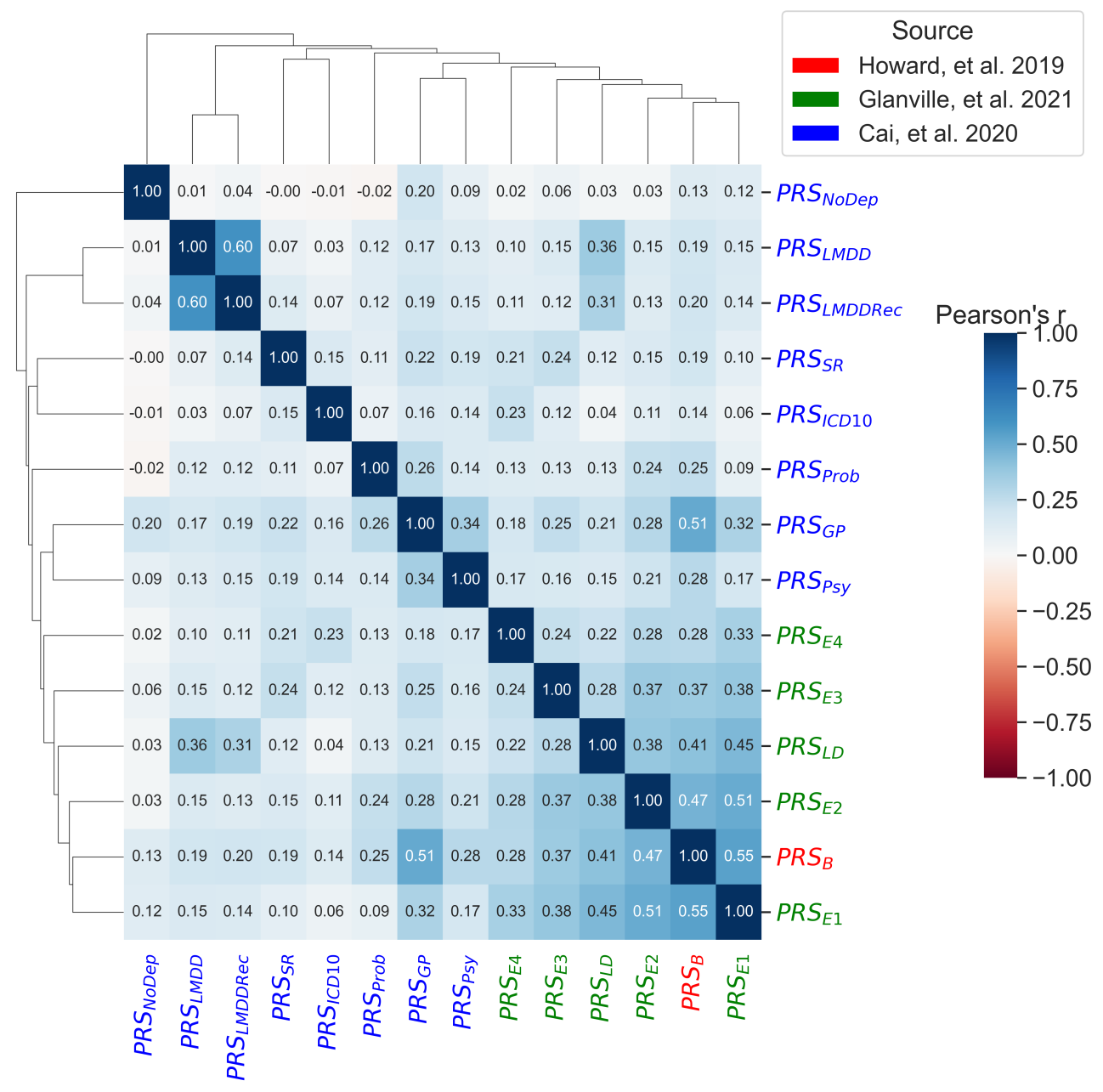


Supplementary Figure S2. Pearson’s correlations including Ward’s clustering, between the PRS for the proposed phenotypic definitions of depression from different sources (Howard, et al. 2019, Glanville, et al. 2021 and Cai, et al. 2020) and definition. PRS_B_, PRS_GP_ (broad) PRS_E1_, PRS_E2_, PRS_E3_, PRS_E4_ (endorsed measures), PRS_LD_, PRS_LMDD_, PRS_LMDDRec_ (Lifetime), PRS_Psy_, PRS_Prob_, PRS_NoDep_ (General practitioner/ Psychiatrist), PRS_SR_ (self-reported), PRS_ICD10_ (ICD). Full description of the phenotype definitions is shown in the supplementary table 2. The diversity in pairwise correlations between the different PRS suggests the presence of phenotype-specific genetic effects, as previously reported by Cai et al. 2020

**
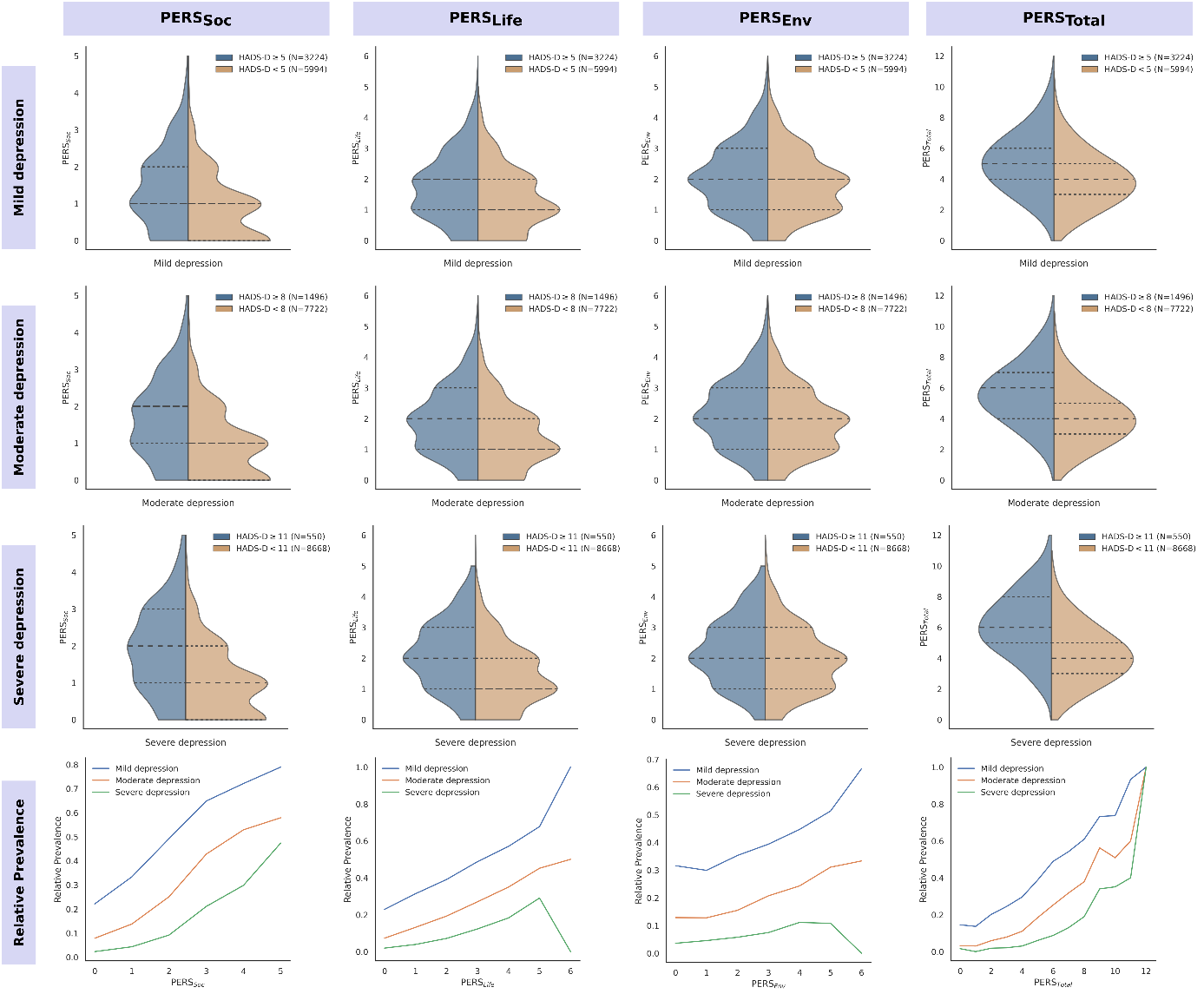
**

Supplementary Figure S3. Split violin plots depicting the differences in PERS social, PERS Lifestyle, and PERS wider environment distributions for all three HADS-D thresholds. Additionally, the relative prevalence shows the proportion of individuals classified as mild, moderate or severe depression given their PERS. Highlighting that socioeconomic factors have a higher effect on depression outcome.

Supplementary Figure S4. Split violin plots depicting the differences in PRS percentile distributions for all three HADS-D thresholds and all PRS for the proposed phenotypic definitions of depression. Additionally, the relative prevalence shows the proportion of individuals classified as mild, moderate or severe depression given their PRS percentile.


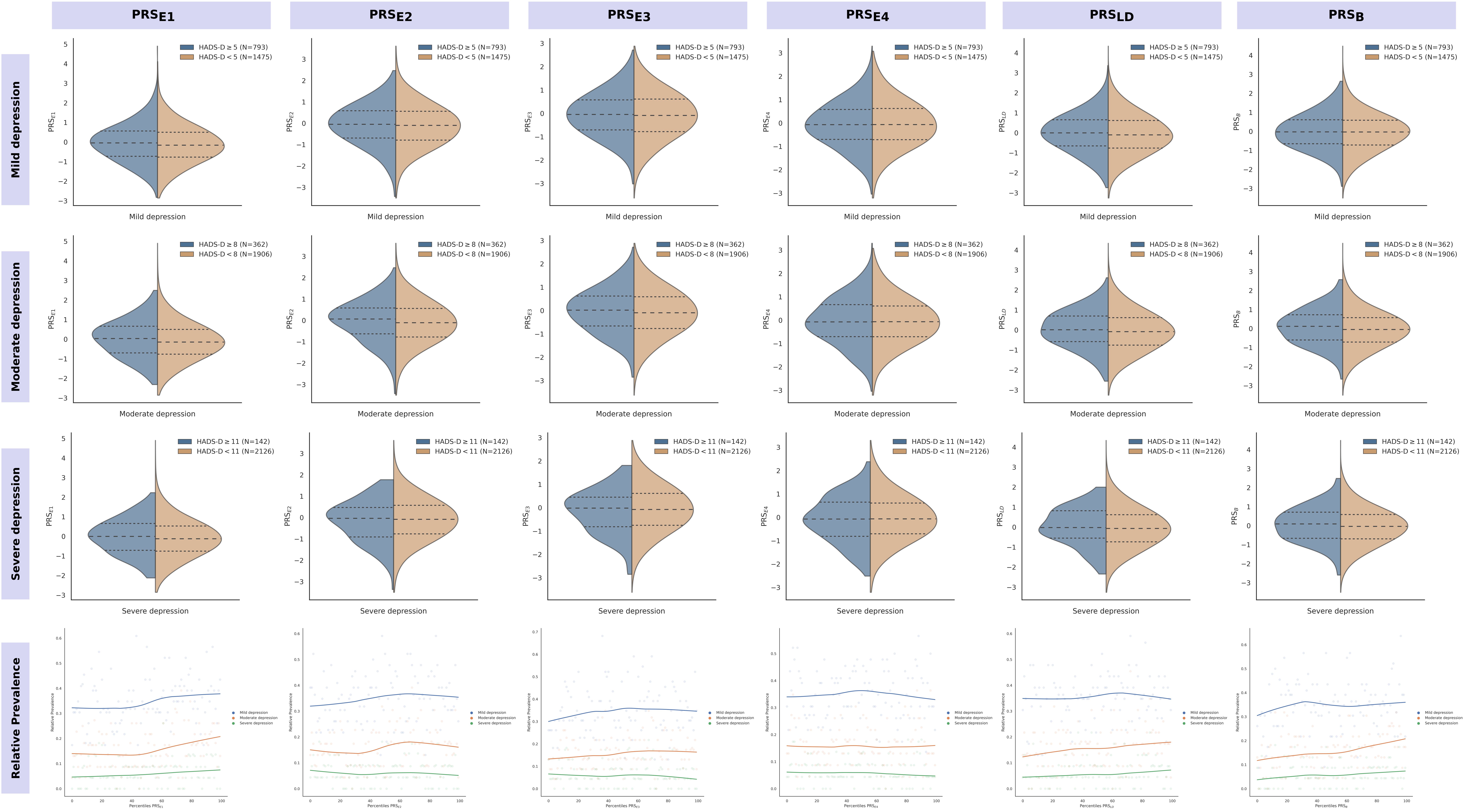




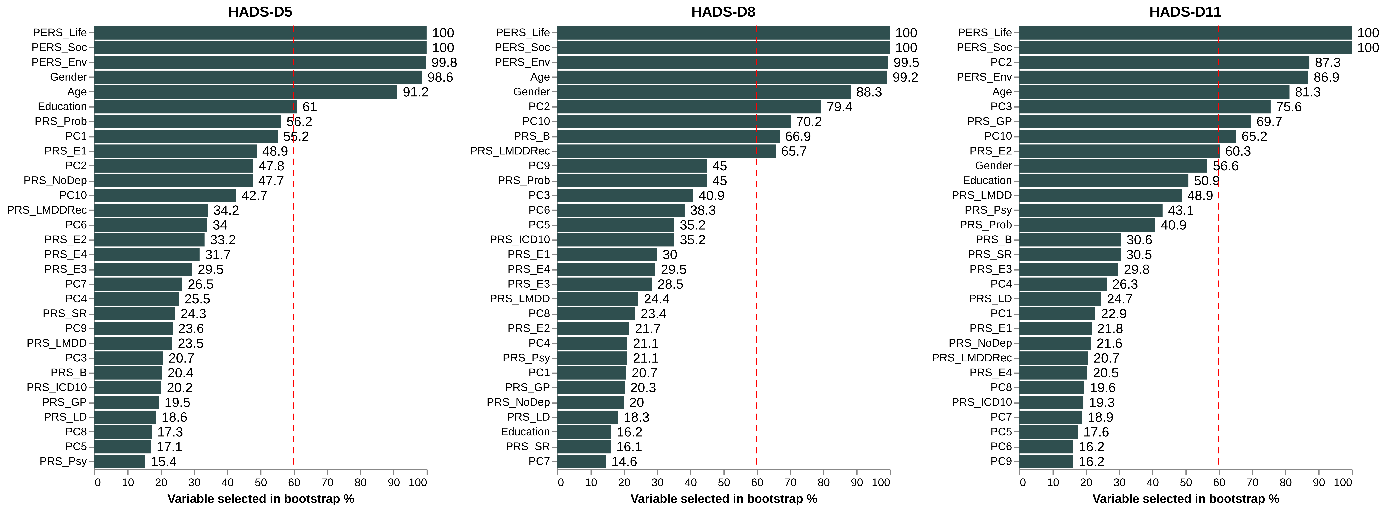


Supplementary Figure S5. Result from the backwards selection of variables using 1000 bootstraps with repetition for each HADS-D threshold, depicting the percentage of models in which each variable was retained in the model. Variables retained in 60% of bootstraps were included in the final model.


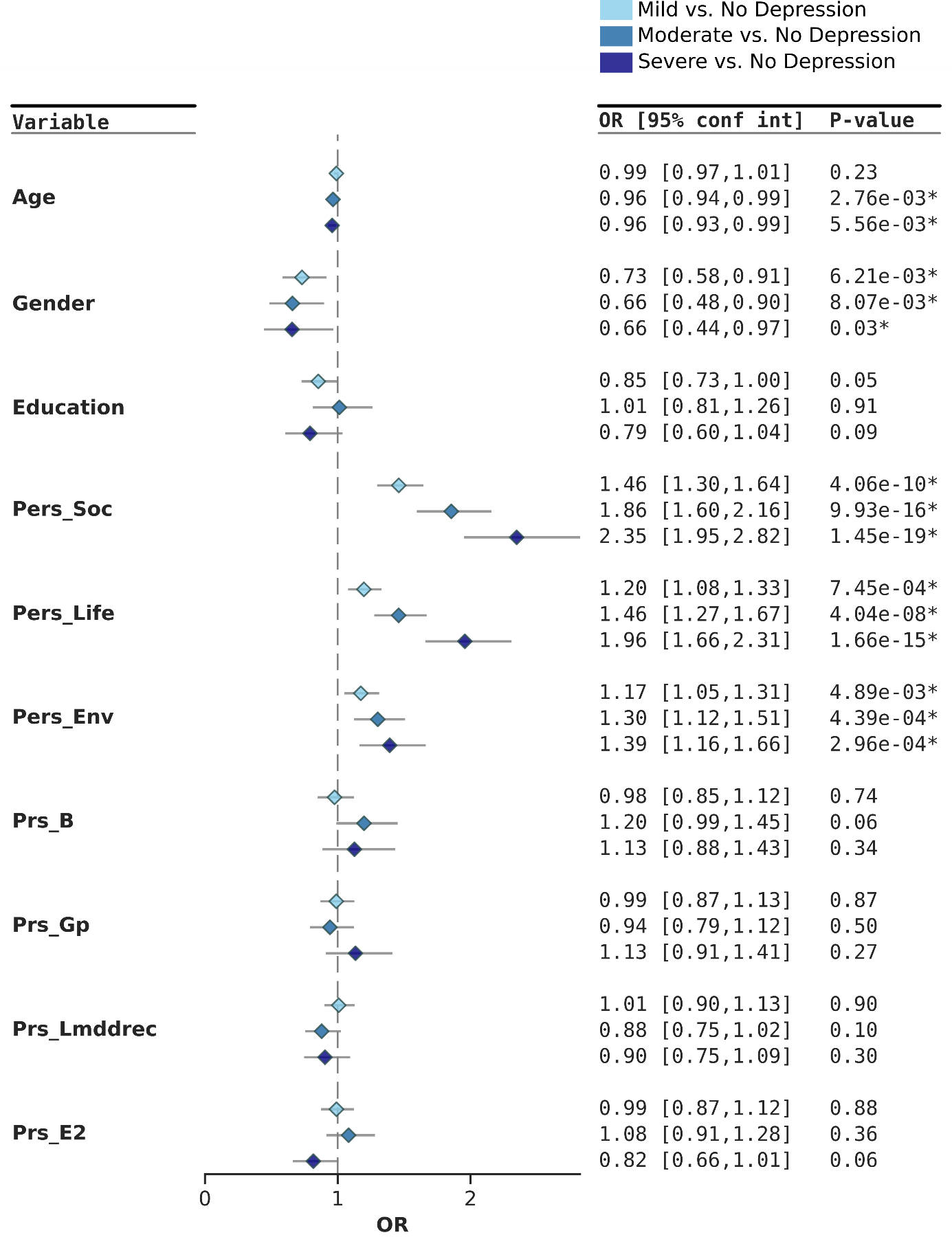


Supplementary Figure S6. Forest plot depicting the results of a multinomial regression including the variables selected in the final models obtained from the backwards elimination of variables for any HADS-D threshold.


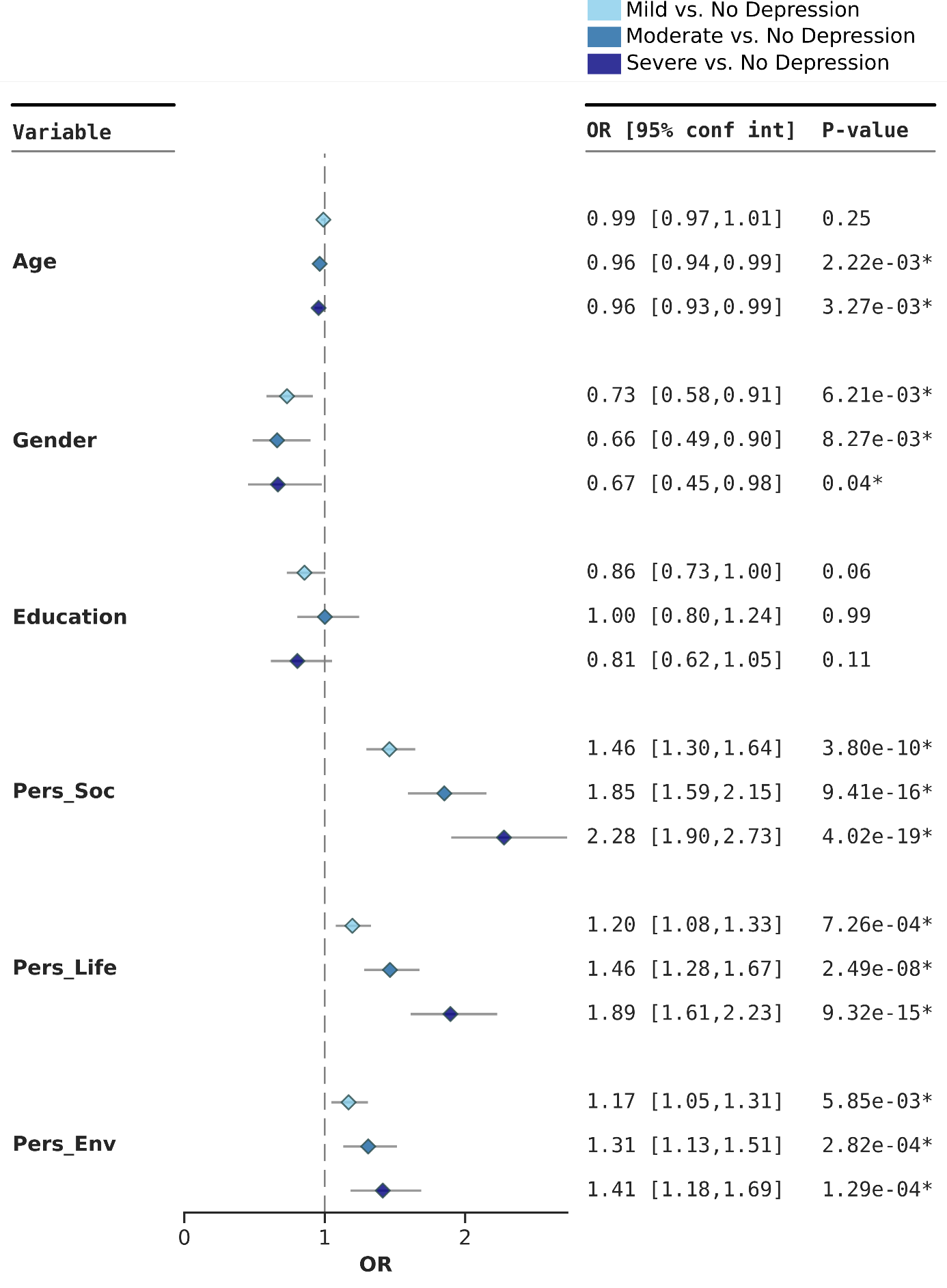


Supplementary Figure S7. Forest plot depicting the results of a multinomial regression including the variables selected in the final model obtained from the backwards elimination of variables for the HADS-D threshold for mild depression.


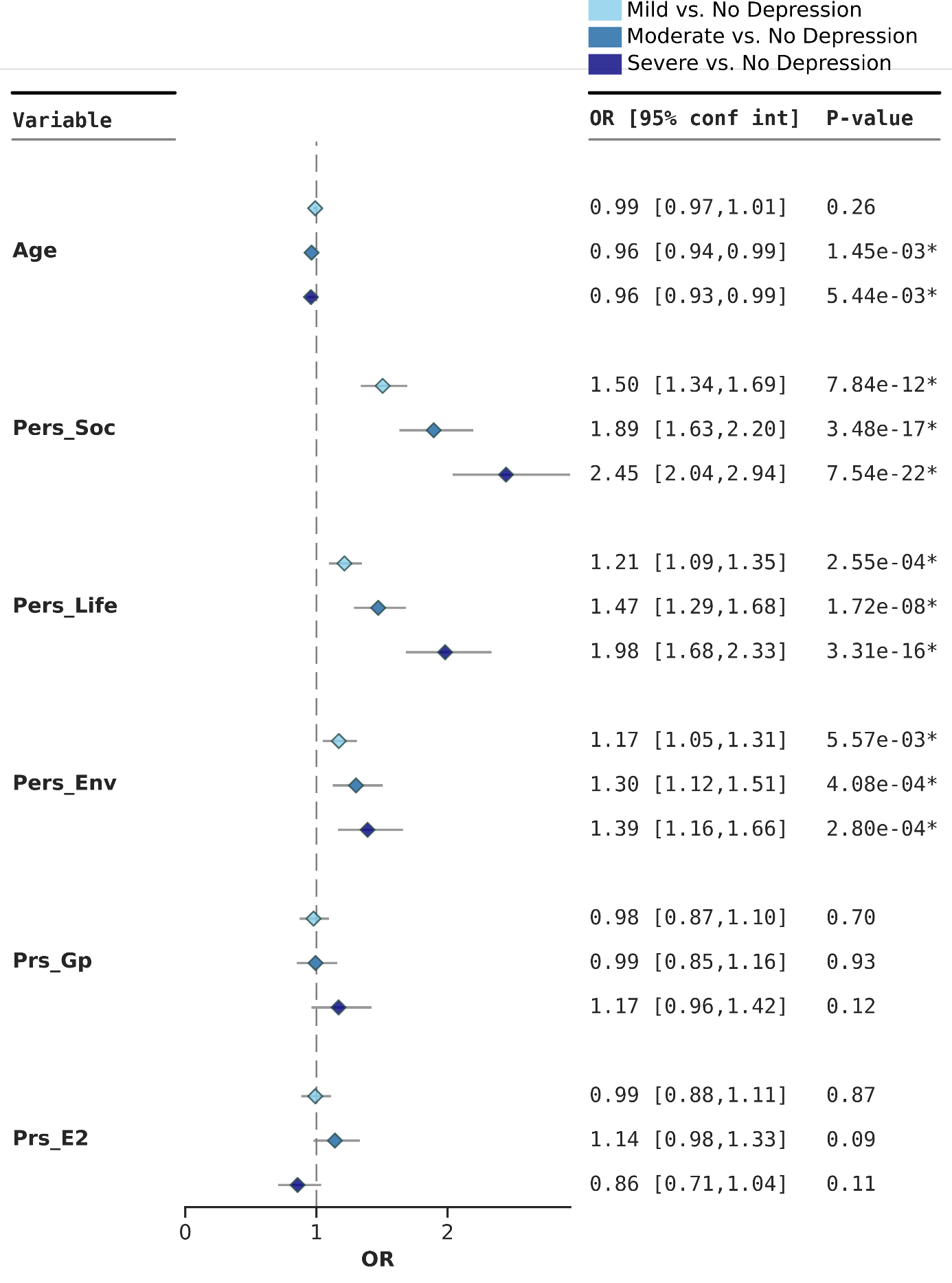


Supplementary Figure S8. Forest plot depicting the results of a multinomial regression including the variables selected in the final model obtained from the backwards elimination of variables for the HADS-D threshold for severe depression.
